# Establishment of a Dual Real-Time PCR Assay for the Identification of African Swine Fever Virus Genotypes I and II in China

**DOI:** 10.3389/fvets.2022.882824

**Published:** 2022-06-01

**Authors:** Qi Gao, Yongzhi Feng, Yunlong Yang, Yizhuo Luo, Ting Gong, Heng Wang, Lang Gong, Guihong Zhang, Zezhong Zheng

**Affiliations:** ^1^Key Laboratory of Zoonosis Prevention and Control of Guangdong Province, College of Veterinary Medicine, South China Agricultural University, Guangzhou, China; ^2^African Swine Fever Regional Laboratory of China, Guangzhou, China; ^3^Research Center for African Swine Fever Prevention and Control, South China Agricultural University, Guangzhou, China; ^4^Maoming Branch, Guangdong Laboratory for Lingnan Modern Agriculture, Maoming, China

**Keywords:** ASFV, dual real-time PCR, *B646L* gene, *E183L* gene, genotypes I and II

## Abstract

Since the first outbreak of ASFV genotype II in China in 2018, ASF has posed a significant threat to the swine industry. After the emergence of genotype I in China in 2020, the epidemic prevention and control have become more difficult. No effective commercial vaccine is currently available, and the disease is difficult to eradicate; therefore, the identification of the ASFV genotype is critical to establish biosafety control measures. In this study, a dual real-time PCR detection method based on *B646L* and *E183L* genes was developed to distinguish between ASFV genotypes I and II by specifically amplifying the genotype I *E183L* gene. The method is strongly specific, detects *B646L* and *E183L* genes simultaneously, and does not cross-react with PEDV, PCV, PRRSV, PRV, and CSFV. The double real-time PCR detection of ASFV genotypes I and II showed a *B646L* amplification curve, and only genotype I showed an *E183L* amplification curve, consistent with our expectations. The method has high sensitivity and the lowest copy numbers detected for recombinant plasmids *B646L* and *E183L* were 1.07 × 10^2^ and 3.13 × 10^4^ copies/μL, respectively. The method is reproducible, and the coefficient of variation for detecting the coefficient of variation (CV) values of the two recombinant plasmids was <2%. Seven samples were positive and 277 were negative, and the results of the two methods were consistent. The dual real-time PCR presented in this study provides a rapid detection method for the identification of ASFV genotypes I and II, which may lead to improving efficient prevention and control measures for ASF in China.

## Introduction

African swine fever (ASF) is an acute, lethal, and highly contagious infectious disease caused by the African swine fever virus (ASFV) in pigs. ASF clinical features are high fever, skin cyanosis, and severe bleeding in lymph nodes and internal organs, with a mortality rate as high as 100% (1). In 1921, ASF was first reported in Kenya, East Africa, and was classified into 24 genotypes based on the sequence of the 3′-end of the *B646L* gene encoding the major capsid protein p72 (2, 3). Multiple ASFV genotypes were circulating in Africa until 1957, when genotype I was first discovered outside Africa, first in Portugal and successively in Europe, South America, Caribbean islands, West Africa, and East and South Africa (4). In 2007, genotype II spread from south-eastern Africa to the Caucasus region of Russia and then became prevalent in more than 30 countries and regions in Europe, Asia, and South America, causing huge economic losses to the swine industry (5, 6).

Since the discovery of ASFV genotype II in China in August 2018 (7), large-scale ASF outbreaks have occurred across the country. ASFV replicates in viral cytoplasmic factories, the presence of viral DNA within the host cell nucleus has been previously reported to be essential for productive infection. Morphological changes of promyelocytic leukemia nuclear bodies (PML-NBs) were found in ASFV-infected swine MDMs, strongly suggesting the viral modulation of cellular antiviral responses and cellular transcription. PML-NBs are involved in interferon (IFN) immune-mediated mechanisms may explain how ASFV interferes with the host immune response (8). In August 2020, our laboratory first detected and isolated ASFV genotype I in pigs. Since then, two ASFVs with genotype I possessing low virulence but high transmission has been reported in China (9). This makes the diagnosis, prevention, and control of ASF more challenging. Therefore, distinguishing between ASFV genotypes I and II is necessary before establishing any strict prevention and control strategies. As no effective vaccine against ASF is currently available in the market, the disease control relies on biosecurity prevention, rapid detection, and culling of infected animals (10). Several molecular and serological methods are currently available to identify animals infected with ASFV. Serological tests are used to determine whether animals have been exposed to ASFV, and molecular tests can detect the presence of ASFV in pigs before they develop clinical symptoms. Polymerase chain reaction (PCR) and enzyme-linked immune sorbent assay are the main routine diagnostic methods for detecting antigens or antibodies (11–14). The World Organization for Animal Health (OIE) recommends the use of validated real-time PCR methods for the diagnosis of ASF (12, 14, 15). Compared to traditional PCR, real-time PCR is fast, highly sensitive, and specific (12, 14). A variety of real-time PCR diagnostic methods for ASFV detection have been developed and validated in the market, most of which target the *B646L* gene (13, 14, 16–18). Studies have shown that, in addition to the *B646L* gene encoding the p72 protein, the tandem repeat sequence (19–21) of the central variable region in the *B602L* and *E183L* genes encoding the p54 protein are also target genes for studying the genotypic diversity of ASFV strains. Combining the *B646L, E183L*, and *pB602L* gene sequences, more data is available to support ASFV typing (19, 22, 23). It has been shown that the *E183L* gene sequence is a valuable candidate for genotyping in molecular epidemiological studies to identify ASFV genotype I (24). Therefore, this study established a dual real-time PCR detection method based on the ASFV *B646L* and *E183L* genes, which can identify the existing ASFV genotypes I and II through two pairs of primers and two probes. The primers and probes of the *B646L* gene were used to determine whether the samples were ASFV-positive, whereas the primers and probes of the *E183L* gene were specific to genotype I and could only detect genotype I-positive samples. Therefore, this method identifies genotypes I and II strains by specifically amplifying the *E183L* gene. The dual real-time PCR detection method presented in this study can provide a powerful tool for the diagnosis of ASFV genotypes I and II strains and could play a key role in the ASF prevention and control in China.

## Materials and Methods

### Viruses, Viral Nucleic Acids, Plasmids, and Clinical Samples

ASFV genotypes I and II nucleic acids, PEDV, PCV2, PRRSV, PRV, CSFV, recombinant plasmids pUC57-B646L, and pCAGGS-E183L were preserved by the Department of Infectious Diseases, School of Veterinary Medicine, South China Agricultural University. The 284 clinical samples were obtained from pig farms in the Guangdong Province and included 178 anticoagulated blood samples, 56 oral swabs, 27 tonsils, and 23 lymph nodes.

### Primers and Probes

The primers and probes used for the *B646L* gene were recommended by China Center for Animal Disease Control (CADC) and the size of the amplified fragment was 159 bp. The primers and probes for the *E183L* gene were designed according to the sequences published in GenBank, using Oigo7 software, in the genotype I specific region, and the size of the amplified fragment was 73 bp. The *B646L* and *E183L* gene GenBank numbers are GZ201801.1 and NC_001659.2, respectively. The *B646L* gene probe 5′-end modified group was FAM, the 3′-end quencher group was MGB; *E183L* gene probe 5′-end modified group was HEX, 3′-end the quencher group was BHQ1, and all primers and probes were synthesized by Thermo Company (Thermo Fisher Scientific, Waltham, MA, USA). Table 1 shows the sequences of the synthesized primers and probes and the sizes of the amplified fragments.

**Table 1:** Primers and probes.

**Name**	**Sequence(5^**′**^-3^**′**^)**	**Amplified fragment size(bp)**
B646L-F	ATAGAGATACAGCTCTTCCAG	159
B646L-R	GTATGTAAGAGCTGCAGAAC	
B646L-Probe	TATCGATAAGATTGAT	
E183L-F	CGCGAGTGCTCATCCGACT	73
E183L-R	GCTTCACAAACAATGTCGGCT	
E183L-Probe	CATCCGACTGAGCCTTACACGACAGTCACT	

### Nucleic Acid Extraction

Tissue samples (0.1 g) were mixed with 1 mL of PBS, ground with a mortar and freeze-thaw three times. Mouth swabs were infiltrated with 1 mL of PBS, and the suspension was centrifuged at 1000 × *g* for 5 min at 4 °C to obtain the supernatant. Nucleic acid extraction from the blood, tissue, and mouth swabs was performed using the Axyprep Body Fluid Viral DNA/RNA Miniprep kit (Axygen, HangZhou, China). The extracted nucleic acid was obtained using the reverse transcription HiScript II 1st Strand cDNA Synthesis kit (+ gDNA wiper) (Vazyme, Nanjing, China) to obtain cDNA, which was stored at−80 °C for further use.

### Dual Real-Time PCR System and Conditions

A real-time PCR 2 × AceQ Universal U kit + Probe Master Mix V2 (Vazyme, Nanjing, China) was used to process the samples, and an ExCycle-48 real-time PCR instrument (GinX, Shanghai, China) was used for double real-time PCR detection and analysis. The amplification conditions used for the reaction is shown in Table 2.

**Table 2:** The amplification reaction conditions.

**Component**	**Volume(μL)**
2 × AceQ Universal U + Probe Master Mix V2	10.0
B646L-F	0.4
B646L-R	0.4
B646L-Probe	0.2
E183L-F	0.4
E183L-R	0.4
E183L-Probe	0.2
Template DNA	2
DEPC H_2_O	up to 20

*PCR program, 37 °C for 2 min; 95 °C for 5 min; 95 °C for 5 min; 95 °C for 10 s, 60 °C for 30 s, 40 cycles*.

### Specific Detection

Nucleic acids were extracted from PEDV, PRRSV, PRV, PCV-2, CSFV, and negative tissues and reverse-transcribed. Double real-time PCR was used to detect ASFV genotypes I and II, PEDV, PRRSV, PRV, PCV-2, CSFV, and negative pig nucleic acid. The primers and probes for double real-time PCR were specifically verified.

### Sensitivity Detection and Construction of a Standard Curve

The concentrations of the recombinant plasmids pUC57-*B646L* and pCAGGS-*E183L* were determined using a NanoDrop Lite spectrophotometer (Thermo Fisher Scientific, Waltham, MA, USA) and were 335.1 and 183.13 ng/μL, respectively. According to the formula to calculate the DNA copy number [dsDNA copy number (copies/μL) = (6.02 × 10^23^(copies/mol) × concentration (ng/μL) × 10^−9^)/DNA length × 660], the copy numbers for the pUC57-*B646L* and pCAGGS-*E183L* plasmids were calculated to be 1.07 × 10^11^ and 3.13 × 10^10^ copies/μL, respectively. A 10-fold serial dilution of the recombinant plasmid was used as a standard template (10^−1^-10^−9^). Three replicates of each dilution were used for real-time PCR detection, sensitivity analysis, and standard curve interpretation.

### Repeated Detection

Different gradient dilutions of the recombinant plasmids pUC57-*B646L* and pCAGGS-*E183L* were used as templates for real-time PCR amplification, and each concentration was repeated three times for repeatability experiments. To verify the repeatability of the established method, the coefficient of variation (CV) was calculated according to the Ct value, CV = (standard deviation SD/mean) × 100%.

## Results

### Dual Real-Time PCR Specific Detection

**Figure 1:**
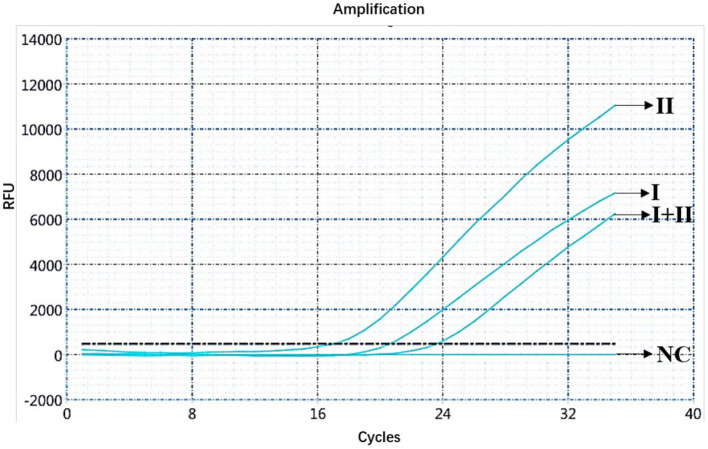
Amplification curve for the FAM channel determined through nucleic acid double fluorescence quantitative PCR of ASFV genotypes I and II, ASFV genotypes I and II mixed samples, and negative pig samples.

**Figure 2:**
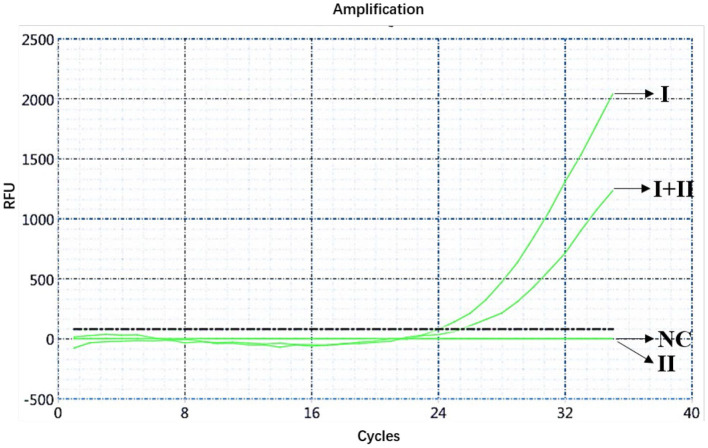
Amplification curve for the HEX channel determined through nucleic acid double fluorescence quantitative PCR of ASFV genotypes I and II, ASFV genotypes I and II mixed samples, and negative pig samples.

The ASFV nucleic acids of genotypes I and II, genotypes I and II mixed samples, and negative pig tissues were determined using double fluorescence quantitative PCR. The FAM channel of the ASFV genotypes I and II and the mixed samples of both genotypes showed amplification curves (Figure 1), while the HEX channel of genotype I and the mixed samples of ASFV genotypes I and II showed amplification curves (Figure 2). This shows that, regardless of the genotype, when ASFV is present, the FAM channel for detecting the B*646L* gene showed an amplification curve. However, only in the presence of genotype I, the HEX channel that detects the *E183L* gene showed an amplification curve, which can be identified as ASFV genotype I.

**Figure 3:**
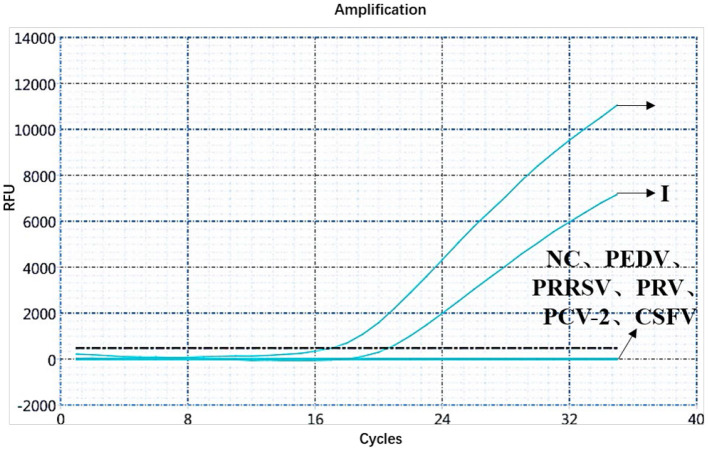
Amplification curve for the FAM channel of ASFV genotypes I and II, PEDV, PRRSV, PRV, PCV-2, CSFV, and negative nucleic acid pig samples determined through dual real-time PCR.

**Figure 4:**
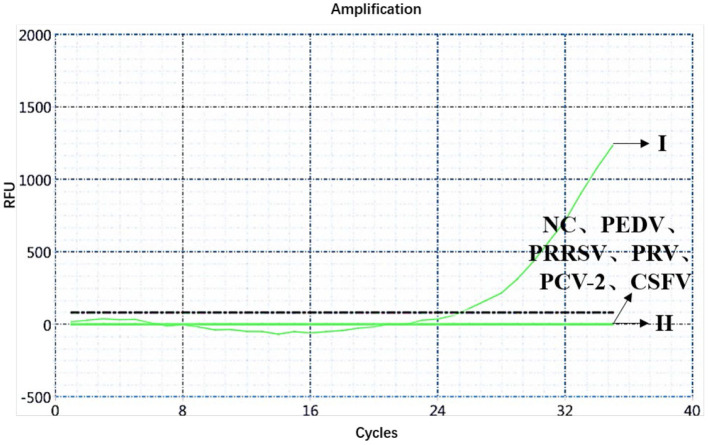
Amplification curve for the HEX channel of ASFV genotypes I and II, PEDV, PRRSV, PRV, PCV-2, CSFV, and negative nucleic acid pig samples determined through dual real-time PCR.

A specificity test of the dual real-time PCR detection was conducted with ASFV genotypes I and II, PEDV, PRRSV, PRV, PCV-2, CSFV, and negative pig nucleic acids as templates. PEDV, PRRSV, PRV, PCV-2, CSFV, and negative pig nucleic acid showed no amplification curve in the FAM and HEX channels, and the result was negative. The FAM channel of ASFV genotypes I and II nucleic acid samples showed amplification curves (Figure 3), and the HEX channel of the ASFV genotype I nucleic acid samples also showed amplification curves (Figure 4). The results indicate that the dual real-time PCR detection method based on *B646L* and *E183L* genes did not cross-react with the nucleic acids of other common porcine-derived viruses and could distinguish the infection of ASFV genotype I strain, which further verified that the method has good specificity.

### Sensitivity Determination of the Dual Real-Time PCR and Establishment of the Standard Curve

**Figure 5:**
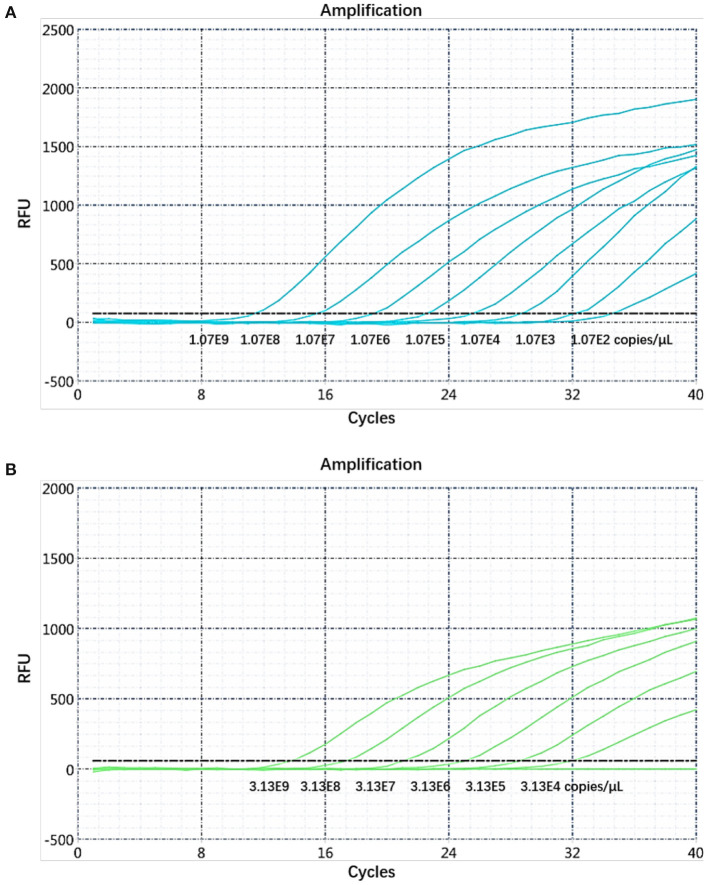
Determination of the dual real-time PCR sensitivity. **(A)** *B646L* gene sensitivity test. **(B)** *E183L* gene sensitivity test.

**Figure 6:**
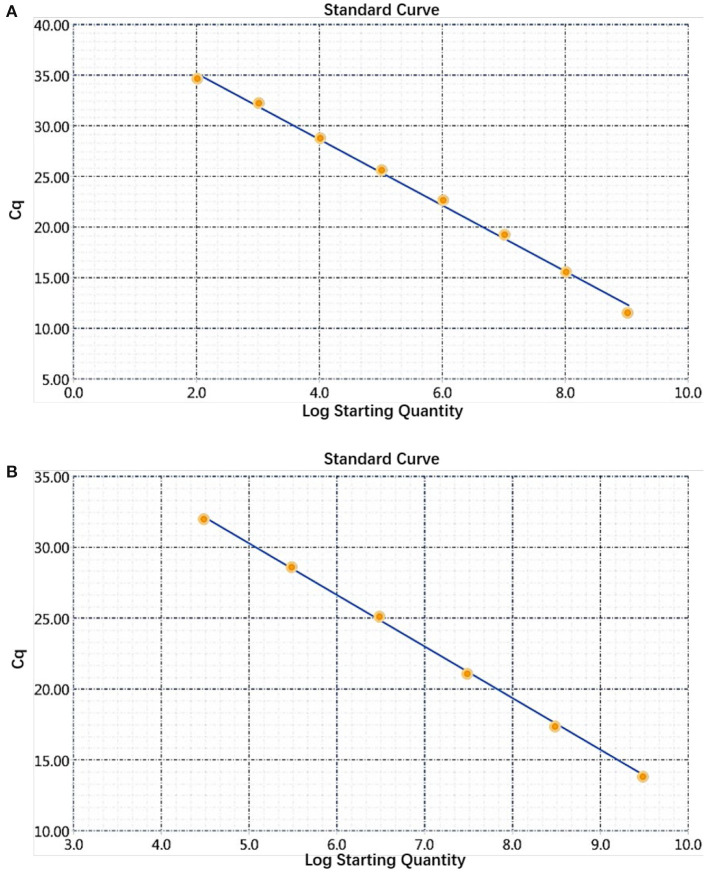
Establishment of a dual real-time PCR standard curve. **(A)** Standard curve for plasmid pUC57-*B646L*, y = −3.258x + 41.69, R^2^ = 0.994. **(B)** Standard curve for plasmid pCAGGS-*E183L*, y = −3.639x + 48.47, R^2^ = 0.997.

The sensitivity of the real-time PCR was determined after 10-fold gradient dilution of the recombinant plasmids pUC57-*B646L* and pCAGGS-*E183L*, and a standard curve was obtained using real-time analysis software. The results showed that the lowest copy numbers for pUC57-*B646L* and pCAGGS-*E183L* determined through real-time PCR were 1.07 × 10^2^ and 3.13 × 10^4^ copies/μL, respectively. This shows that the primers and probes used for this method detected both genes with high sensitivity (Figure 5). The slopes of the standard curves for the two plasmids, pUC57-*B646L* and pCAGGS-*E183L*, were −3.258 and −3.639, respectively, and the R^2^ values of the standard curves were 0.994 and 0.997, respectively (Figure 6), indicating that each diluted sample showed a good linear relationship.

### Repeatability and Reproducibility of the Dual Real-Time PCR

Repeatability and reproducibility experiments were performed using 10-fold serial dilutions of the recombinant plasmids, pUC57-*B646L* and pCAGGS-*E183L*, and calculated according to the Ct values of the results. The intra-assay CV for pUC57-*B646L* was 0.40–1.88% (Table 3) and the intra-assay CV for pCAGGS-*E183L* was 0.42–1.14% (Table 4). The inter-assay CV of pUC57-*B646L* was 0.441.86% (Table 3) and the inter-assay CV of pCAGGS-*E183L* was 0.56–1.19% (Table 4). The CV values were all <2%, indicating that the method has good repeatability.

**Table 3:** Intra-reproducibility and intra-repeatability of *B646L* gene by dual real-time PCR.

**Template dilution**	**Ct value**	**Average value**	**Standard deviation**	**CV (%)**	**Ct value**	**Average value**	**Standard deviation**	**CV (%)**
	11.44				11.26			
10^−2^	11.82	11.69	0.18	1.57	11.54	11.49	0.21	1.86
	11.56				11.68			
	15.47				15.74			
10^−3^	15.72	15.53	0.27	1.73	15.53	15.56	0.17	1.07
	15.34				15.41			
	19.15				19.26			
10^−4^	19.29	19.47	0.25	1.27	19.65	19.37	0.25	1.28
	19.64				19.19			
	22.56				22.67			
10^−5^	22.89	22.59	0.42	1.88	22.91	22.72	0.17	0.73
	22.29				22.59			
	25.53				25.34			
10^−6^	25.23	25.5	0.39	1.52	25.72	25.54	0.19	0.75
	25.78				25.56			
	28.69				28.43			
10^−7^	28.29	28.43	0.20	0.70	28.65	28.46	0.18	0.64
	28.57				28.29			
	32.15				32.67			
10^−8^	32.06	32.15	0.13	0.40	32.25	32.40	0.23	0.72
	32.24				32.29			
	34.57				34.99			
10^−9^	34.98	34.87	0.16	0.45	35.08	34.95	0.15	0.44
	34.76				34.78			

**Table 4:** Intra-reproducibility and intra-repeatability of *E183L* gene by dual real-time PCR/.

**Template dilution**	**Ct value**	**Average value**	**Standard deviation**	**CV (%)**	**Ct value**	**Average value**	**Standard deviation**	**CV (%)**
	13.75				13.69			
10^−1^	13.68	13.57	0.16	1.14	13.42	13.61	0.16	1.19
	13.46				13.71			
	17.29				17.52			
10^−2^	17.58	17.68	0.14	0.80	17.38	17.54	0.18	1.00
	17.78				17.73			
	21.01				21.11			
10^−3^	21.54	21.43	0.16	0.73	21.45	21.39	0.26	1.19
	21.32				21.61			
	25.04				25.28			
10^−4^	25.56	25.70	0.19	0.74	25.49	25.50	0.22	0.86
	25.83				25.72			
	28.53				28.41			
10^−5^	28.03	28.25	0.33	1.08	28.32	28.28	0.16	0.56
	28.46				28.10			
	31.92				32.01			
10^−6^	32.45	32.36	0.13	0.42	32.54	32.28	0.27	0.82
	32.26				32.29			

### Analysis of Clinical Samples

**Figure 7:**
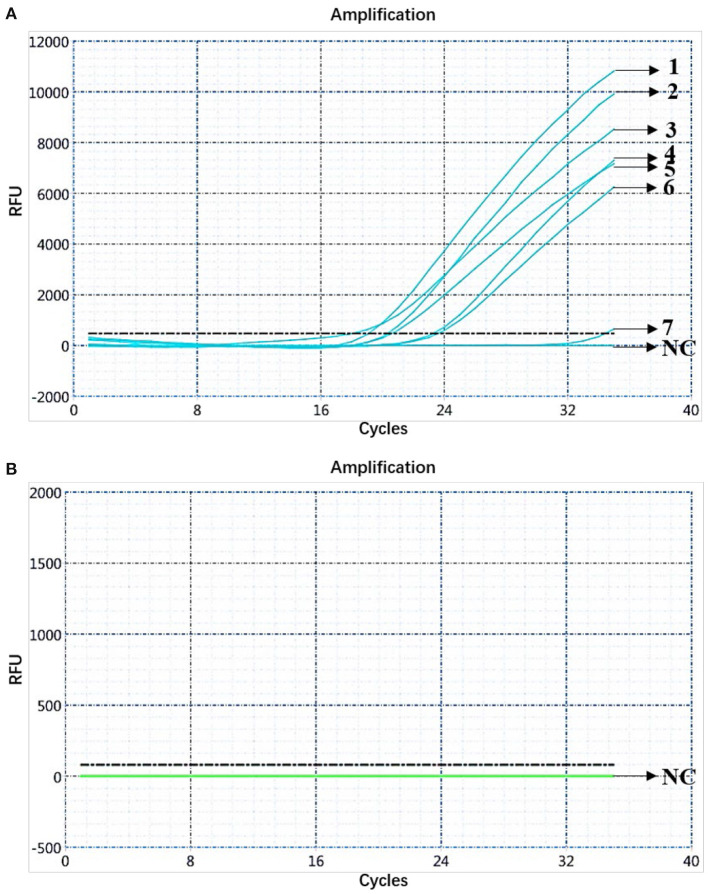
Analysis of clinical samples. **(A)** Amplification curve for the FAM channel determined through nucleic acid dual fluorescence quantitative PCR of pig clinical samples (284). Pig clinical samples (284) were analyzed using dual real-time PCR; seven samples were ASFV-positive. **(B)** Amplification curve for the HEX channel determined through nucleic acid dual fluorescence quantitative PCR of pig clinical samples (284) and the remaining 277 samples were negative.

Clinical samples [284] from pigs from farms in the Guangdong Province were analyzed using the dual fluorescent quantitative PCR. The analysis showed 7 samples (1–7) to be positive for ASFV *B646L* and *E183L* genes, and the remaining 277 samples to be negative, which was consistent with the results of the fluorescence quantitative PCR method recommended by the OIE (Figure 7). Then, the samples were subjected to high-temperature and high-pressure treatments according to biosafety operation specifications. Among the seven ASFV-positive samples, only the FAM channel showed the amplification curve of the *B646L* gene, indicating that the seven positive samples were presented genotype II infection (Figure 7). The seven positive samples were collected from three ASFV-infected pigs from the Xiangzhou District, Zhuhai City, Guangdong Province. The information was published by the Ministry of Agriculture and Rural Affairs of the People's Republic of China: http://www.moa.gov.cn/gk/yjgl_1/yqfb/201812/t20181219_6165233.htm. Nucleic acids were prepared and preserved at the National African Swine Fever Regional Laboratory (Guangzhou, China).

## Discussion

ASF is a high-mortality and economically important viral disease of domestic and wild boars that causes acute hemorrhagic fever, with a fatality rate of up to 100% (5, 25). ASFV was first reported in Kenya 100 years ago, and today ASF poses a great threat to the swine industry worldwide; however, the clinical symptoms and case fatality rates of ASF change as the disease progresses (26). After the discovery of the acutely infective ASFV genotype II in China in 2018, due to imperfect prevention and control measures, ASFV genotype I, with lower mortality and stronger transmission ability, too was discovered in 2020. Both low virulent genotype II and new-emerging genotype I ASFVs showed lower pathogenicity but high transmissibility, which caused chronic and persistent infections in pigs. Pigs infected with these low virulent ASFV strains have milder clinical symptoms but constantly shed via oral and rectal routes at a low level, which poses a great challenge for the early diagnosis and control of the disease (9). As China is a big country of pig raising, the epidemic of ASF has brought a serious threat to the development of China's animal husbandry and the economic stability of the pig industry. Therefore, the prevention and control of ASF is the top priority of my country's animal husbandry. Epidemiological studies have shown that the entry of ASFV into non-ASF areas is mainly related to the construction of international airports and use of contaminated seaport waste to feed pigs (19). Combined with extensive commercial trade, ASF-free countries are always at risk of ASFV being introduced into their territories. As there is currently no efficient commercial vaccine for ASF, control and eradication strategies are mainly based on the early screening and implementation of strict biosecurity prevention and control measures. Therefore, laboratories must have rapid and sensitive detection procedures, and the identification of strain genotypes is critical for ASF control and eradication. It has been reported that part of the gene encoding the p72 protein and the *E183L* gene encoding the structural protein p54 can be used to identify the ASFV genotype. By combining the sequences of these two genes, a high-resolution method for identifying the virus genotype can be established (27). In this study, as ASFV genotypes I and II strains are prevalent in China, a novel dual fluorescence quantitative PCR detection method based on *B646L* and *E183L* genes was developed to identify these ASFV genotypes. If only the *B646L* gene is detected, it means that the test sample is positive for ASFV genotype II; if the *B646L* and *E183L* genes are detected, it means that the test sample is positive for genotype I. It is worth noting that only when the *B646L* gene is detected, the test sample can be considered ASFV positive; otherwise, it is considered negative. The specific detection of this method showed that the primers and probes used did not cross-react with other swine-derived viruses commonly found in pig farms, and the lowest copy numbers determined for *B646L* and *E183L* were 1.07 × 10^2^ and 3.13 × 10^4^ copies/μL, respectively. The standard curve presented a good linear relationship, and the experimental results were true and reliable. At the same time, 284 clinical swine samples were analyzed using the dual real-time PCR detection method, and seven ASFV-positive samples were found, which was consistent with the detection method recommended by the OIE. Therefore, the use of this detection method provides a powerful tool for identifying the ASFV genotypes strains in China to assess the origin and transmission trajectory of ASF outbreaks more accurately in epidemic areas and establish a solid foundation for ASF epidemiological investigation and epidemic prevention and control.

## Data Availability Statement

The original contributions presented in the study are included in the article/supplementary material, further inquiries can be directed to the corresponding authors.

## Author Contributions

QG and ZZ designed the study. QG and YF were involved in the acquisition of data, analysis, figure preparation, and supervised the study. YY and HW contributed to some of the laboratory experiments and data analysis. YL and TG helped revise the manuscript. QG drafted the original paper. All authors read and approved the final manuscript.

## Funding

This work was supported by the Key-Area Research and Development Program of Guangdong Province (grant number 2019B020211003), the National Natural Science Foundation of China (grant number 31941004), the Science and Technology Project of Guangdong Pig Industrial Park (grant number GDSCYY2020-024), and the China Agriculture Research System of MOF and MARA (grant number car35).

## Conflict of Interest

The authors declare that the research was conducted in the absence of any commercial or financial relationships that could be construed as a potential conflict of interest.

## Publisher's Note

All claims expressed in this article are solely those of the authors and do not necessarily represent those of their affiliated organizations, or those of the publisher, the editors and the reviewers. Any product that may be evaluated in this article, or claim that may be made by its manufacturer, is not guaranteed or endorsed by the publisher.
